# RNF: a general framework to evaluate NGS read mappers

**DOI:** 10.1093/bioinformatics/btv524

**Published:** 2015-09-09

**Authors:** Karel Břinda, Valentina Boeva, Gregory Kucherov

**Affiliations:** ^1^LIGM/CNRS, Université Paris-Est, 77454 Marne-la-Vallée, France,; ^2^Inserm, U900, Bioinformatics, Biostatistics, Epidemiology and Computational Systems Biology of Cancer, 75248 Paris, France,; ^3^Institut Curie, Centre de Recherche, 26 rue d’Ulm, 75248 Paris, France and; ^4^Mines ParisTech, 77300 Fontainebleau, France

## Abstract

**Motivation:** Read simulators combined with alignment evaluation tools provide the most straightforward way to evaluate and compare mappers. Simulation of reads is accompanied by information about their positions in the source genome. This information is then used to evaluate alignments produced by the mapper. Finally, reports containing statistics of successful read alignments are created.

In default of standards for encoding read origins, every evaluation tool has to be made explicitly compatible with the simulator used to generate reads.

**Results:** To solve this obstacle, we have created a generic format Read Naming Format (Rnf) for assigning read names with encoded information about original positions. Futhermore, we have developed an associated software package RnfTools containing two principal components. MIShmash applies one of popular read simulating tools (among DwgSim, Art, Mason, CuReSim, etc.) and transforms the generated reads into Rnf format. LAVEnder evaluates then a given read mapper using simulated reads in Rnf format. A special attention is payed to mapping qualities that serve for parametrization of Roc curves, and to evaluation of the effect of read sample contamination.

**Availability and implementation: **RnfTools: http://karel-brinda.github.io/rnftools Spec. of Rnf: http://karel-brinda.github.io/rnf-spec

**Contact:** karel.brinda@univ-mlv.fr

## 1 Introduction

The number of Next-Generation Sequencing (Ngs) read mappers has been rapidly growing during the last years. Then, there is an increasing demand of methods for evaluation and comparison of mappers to select the most appropriate one for a specific task. The basic approach to compare mappers is based on simulating Ngs reads, aligning them to the reference genome and assessing read mapping accuracy using a tool evaluating if each individual read has been aligned correctly.

There exist many read simulators [Art (Huang *et al.*, 2011), CuReSim (Caboche *et al.*, 2014), DnEmulator (Frith *et al.*, 2012), DwgSim (http://github.com/nh13/dwgsim), FastqSim (Shcherbina, 2014), FlowSim (Balzer *et al.*, 2010), GemSim (McElroy *et al.*, 2012), Mason (Holtgrewe, 2010), PbSim (Ono *et al.*, 2013), Pirs (Xu *et al.*, 2012), SINC (Pattnaik *et al.*, 2014), WgSim (http://github.com/lh3/wgsim), Xs (Pratas *et al.*, 2014)] as well as many evaluation tools [CuReSimEval, DwgSim_Eval, RABeMa (Holtgrewe *et al.*, 2011), Seg-Suite (http://cbrc3.cbrc.jp/∼martin/seg-suite/), WgSim_Eval, etc.]. However, each read simulator encodes information about the origin of reads in its own manner. This makes combining tools complicated and makes writing ad-hoc conversion scripts inevitable.

Here we propose a standard for naming simulated Ngs reads, called Read Naming Format (Rnf), that makes evaluation tools for read mappers independent of the tool used for read simulation. Furthermore, we introduce RnfTools, an easily configurable software, to obtain simulated reads in Rnf format using a wide class of existing read simulators, and also to evaluate Ngs mappers.

### 1.1 Simulation of reads

A typical read simulator introduces mutations into a given reference genome (provided usually as a Fasta file) and generates reads as genomic substrings with randomly added sequencing errors. Different statistical models can be employed to simulate sequencing errors and artefacts observed in experimental reads. The models usually take into account CG-content, distributions of coverage, of sequencing errors in reads and of genomic mutations. Simulators can often learn their parameters from an experimental alignment file.

At the end, information about origin of every read is encoded in some way and the reads are saved into a Fastq file.

### 1.2 Evaluation of mappers

When simulated reads are mapped back to the reference sequence and possibly processed by an independent post-processing tool (remapping around indels, etc.), an evaluation tool inputs the final alignments of all reads, extracts information about their origin and assesses if every single read has been aligned to a correct location (and possibly with correct edit operations). The whole procedure is finalized by creating a summarizing report.

Various evaluation strategies can be employed (see, e.g. introduction of Caboche *et al.*, 2014). Final statistics usually strongly depend on the definition of a correctly mapped read, mapper’s approach to deal with multi-mapped reads and with mapping qualities.

### 1.3 Existing read naming approaches

Depending on the read simulator, information about the read’s origin is either encoded in its name, or stored in a separate file, possibly augmented with information about the expected read alignment. While WgSim encodes the first nucleotide of each end of the read in the read name, DwgSim and CuReSim encode the leftmost nucleotide of each end. Unfortunately, these read naming schemes were specifically designed for particular sequencing technologies and single evaluation strategies, therefore they are not suitable as generic formats. Art produces Sam and Aln alignment files, Mason creates Sam files and Pirs makes text files in its own format.

## 2 Methods

We have created Rnf, a standard for naming simulated reads. It is designed to be robust, easy to adopt by existing tools, extendable, and to provide human-readable read names. It respects a wide range of existing sequencing technologies as well as their possible future evolution (e.g. technologies producing several ‘subreads’). We then developed a utility for generating Rnf-compliant reads using existing simulators, and an associated mapper evaluation tool.

### 2.1 Read naming format (RNF)

#### 2.1.1 Read tuples

*Read tuple* is a tuple of sequences (possibly overlapping) obtained from a sequencing machine from a single fragment of DNA. Elements of these tuples are called *reads.* For example, every ‘paired-end read’ is a *read tuple* and both of its ‘ends’ are individual *reads* in our notation.

To every *read tuple*, two strings are assigned: a short read name (SRN) and a long read name (LRN). SRN contains a hexadecimal unique ID of the *tuple* prefixed by ’#’. LRN consists of four parts delimited by double-underscore: (i) a prefix (possibly containing expressive information for a user or a particular string for sorting or randomization of order of *read tuples*), (ii) a unique ID, (iii) information about origins of all segments (see below) that constitute *reads* of the *tuple*, (iv) a suffix containing arbitrary comments or extensions (for holding additional information). Preferred final read names are LRNs. If an LRN exceeds 255 (maximum allowed read length in Sam), SRNs are used instead and a SRN–LRN correspondence file must be created.

#### 2.1.2 Segments

**Fig. 1.**
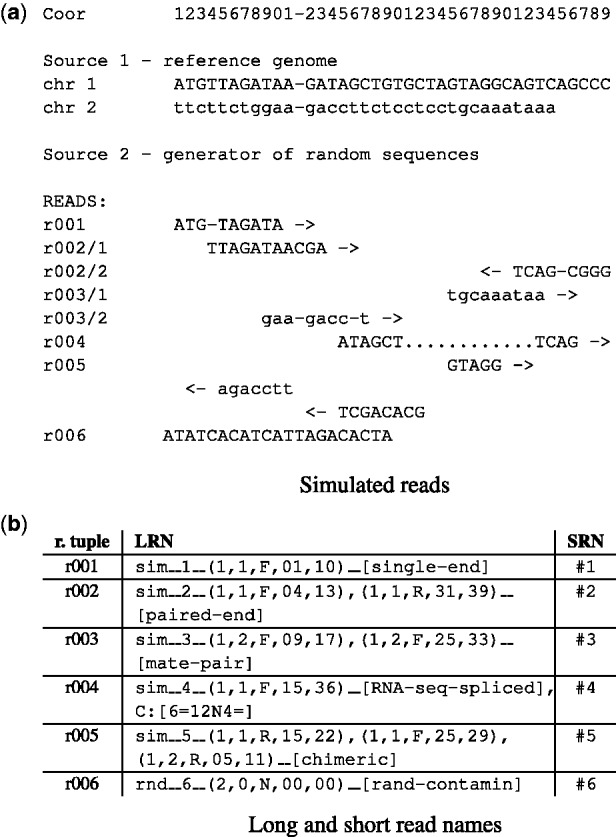
Examples of simulated reads (in our definition *read tuples*) and their corresponding Rnf names, which can be used as read names in the final Fastq file: a single-end read (r001); a paired-end read (r002); a mate-pair read (r003); a spliced RNA-seq read (r004); a chimeric read (r005); and a random contaminating read with unspecified coordinates (r006)

*Segments* are substrings of a *read* which are spatially distinct in the reference and correspond to individual lines in a Sam file. Since spliced RNA-seq *reads* (Fig. 1, r004) are usually reported in single lines in SAM, we recommend to keep them in single Rnf segments without splitting even though they might be considered spatially distinct. Thus, each *read* has an associated chain of *segments* and we associate a *read tuple* with *segments* of all its *reads.*

Within our definition, a ‘single-end read’ (Fig. 1, r001) consists of a single *read* with a single *segment* unless it comes from a region with genomic rearrangement. A ‘paired-end read’ or a ‘mate-pair read’ (Fig. 1, r002 and r003) consists of two *reads*, each with one *segment* (under the same condition). A ‘strobe read’ consists of several *reads.* Chimeric *reads* (i.e. reads corresponding to a genomic fusion, a long deletion, or a translocation; Fig. 1, r005) have at least two *segments.*

For each *segment*, the following information is encoded: leftmost and rightmost 1-based coordinates in its reference, ID of its reference genome, ID of the chromosome and the direction (‘F’ or ‘R’). The format is:

(genome_id,chromosome_id,direction, L_coor,R_coor).

*Segments* in LRN are recommended to be sorted with the following keys: source, chromosome, L_coor, R_coor, direction. When some information is not available (e.g. the rightmost coordinate), zero is used (‘N’ in case of direction; Fig. 1, r006).

#### 2.1.3 Extensions

The basic standard can be extended for specific purposes by extensions. They are part of the suffix and encode supplementary information (e.g. information about CIGAR strings, sequencing errors, or mutations).

### 2.2 RNFtools

We also developed RnfTools, a software package associated with Rnf. It has two principal components: MIShmash for read simulation and LAVEnder for evaluation of NGS read mappers. RnfTools has been created using SnakeMake (Köster and Rahmann, 2012), a Python-based Make-like build system. All employed external programs are installed automatically when needed. The package also contains a lightweight console tool rnftools which can, in addition, be used for conversion of existing data and transformation of RNF coordinates using a LiftOver chain file.

MIShmash is a pipeline for simulating reads using existing simulators and combining obtained sets of reads together (e.g. to simulate contamination or metagenomic samples). Its output files respect Rnf format, therefore, any Rnf-compatible evaluation tool can be used for evaluation.

**Fig. 2.**
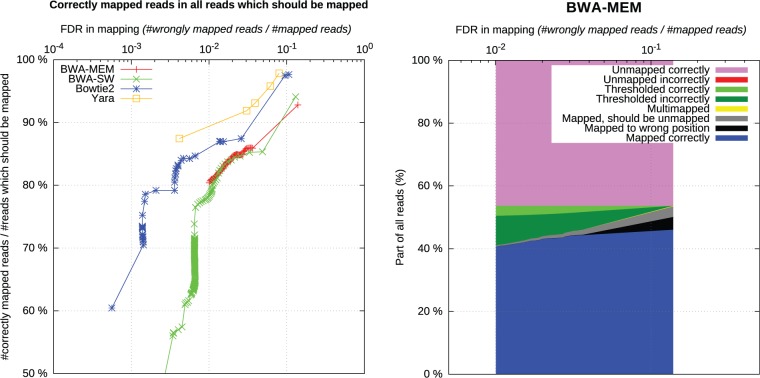
Example of two graphs produced by LAVEnder as a part of comparison of mapper capabilities of contamination detection. 200.000 single-end reads were simulated from human and mouse genomes (100.000 from HG38, 100.000 from MM10) by DwgSim using MIShmash and mapped to HG38. All LAVEnder graphs have false discovery rate on *x*-axis and use mapping quality as the varying parameter for plotted curves. This experiment reveals that YARA copes with contamination better than Bowtie2, BWA-MEM and BWA-SW

LAVEnder is a program for evaluating mappers. For a given set of Bam files, it creates an interactive Html report with several graphs. In practice, mapping qualities assigned by different mappers to a given read are not equal (although mappers tend to unify this). Moreover, even for a single mapper, mapping qualities are very data-specific. Therefore, results of mappers after the same thresholding on mapping quality are not comparable. To cope with this, we designed LAVEnder to use mapping qualities as parameterization of curves in ‘sensitivity-precision’ graphs (like it has been done in Li (2013)). Examples of output of LAVEnder can be found in Figure 2.

## 3 Conclusion

We designed Rnf format and propose it as a general standard for naming simulated Ngs reads. We developed RnfTools consisting of MIShmash, a pipeline for read simulation, and LAVEnder, an evaluation tool for mappers, both following the Rnf convention (thus inter-compatible). Currently, MIShmash has a built-in interface with the following existing read simulators: Art, CuReSim, DwgSim, Mason and WgSim.

We expect that authors of existing read simulators will adopt Rnf naming convention as it is technically simple and would allow them to extend the usability of their software. We also expect authors of evaluation tools to use Rnf to make their tools independent of the used read simulator.
